# Nursing Students’ Knowledge and Motivations Regarding Blood Donation Following the COVID-19 Pandemic in Hong Kong: A Cross-Sectional Study

**DOI:** 10.3390/healthcare14050610

**Published:** 2026-02-27

**Authors:** Maria Shuk Yu Hung, Grace Sun King Wan, Yuk Ting Lau

**Affiliations:** S.K. Yee School of Health Sciences, Saint Francis University, Hong Kong, Chinashandylauyt@gmail.com (Y.T.L.)

**Keywords:** blood donation, knowledge, motivations, nursing students, COVID-19, Hong Kong

## Abstract

**Background/Objectives:** Blood transfusions save lives and improve health, but there has been a global blood shortage and a significant disparity in blood donation worldwide in recent years. As future healthcare professionals who educate and promote blood donation, the knowledge and attitude of nursing students are paramount to improving future motivation and engagement. In this study, we aimed to investigate the knowledge and motivations of nursing students regarding blood donation following the COVID-19 pandemic in Hong Kong. **Methods:** With the cross-sectional descriptive design of this study, we used a well-validated Blood Donor Identity Survey (Chinese version), as well as convenience sampling of university students aged ≥18 enrolled in full-time nursing programs at a large university. Ethical approval was sought prior to data collection in November 2023. A total of 711 of the ~2200 target participants returned the questionnaires, with 650 completing them (29.5%). **Results:** Of these 650, 465 (71.5%) were non-blood donors and 185 (28.5%) were blood donors, with blood donors (9.77 ± 1.28) demonstrating a significantly higher total knowledge score than non-blood donors (9.46 ± 1.33), with *p* = 0.006. Prior experience of receiving blood (OR = 5.81, 95% CI: 2.92–11.54, *p* < 0.001), older age (OR = 1.22, 95% CI: 1.12–1.33, *p* < 0.001), knowing someone who had donated blood (OR = 2.63, 95% CI: 1.29–5.35, *p* = 0.008), and having a religious affiliation (OR = 1.81, 95% CI: 1.07–3.06, *p* = 0.028) were found to be significant factors associated with a greater willingness to donate blood. Conversely, taking medication was found to be a significant factor associated with a lower likelihood of being a blood donor (OR = 0.24, 95% CI: 0.09–0.63, *p* = 0.004). Within the ratings of donor identity, higher Amotivation scores reduced the odds of blood donation (OR = 0.83, 95% CI: 0.78–0.89, *p* < 0.001), while an increased score on Identified Regulation was significantly and positively related to donor status (OR = 1.15, 95% CI: 1.09–1.22, *p* < 0.001). **Conclusions:** Blood donation rates among local nursing students were low after the pandemic, despite moderate overall knowledge. Collaboration among the Hong Kong government, healthcare organizations, and university nursing faculties is vital to promote blood donation among future nursing professionals and the public.

## 1. Introduction

The World Health Organization emphasizes the importance of safe and adequate blood transfusion for lifesaving and improving health [1]. Even with the numerous advancements in medical technology, there is still no artificial replacement for blood. The ongoing, unforeseeable demand for blood products depends on a consistent, dependable group of voluntary donors who can replenish and maintain sufficient blood availability [2]. Nearly half of all annual blood donations originate from high-income countries, contributed by various types of donors [1,3]. However, there is a significant disparity in blood donation rates globally, with 31.5, 23, and five donations per 1000 people from high-, middle-, and low-income countries, respectively [1]. In 2019, Roberts et al. reported that 119 of 195 countries had an insufficient blood supply, including China [4]. Furthermore, about one-third (33%) of blood donations worldwide are from females, and more young adults donate blood in low- and middle-income countries than in high-income countries [1]. Recruiting young adults as blood donors is vital to ensuring a blood supply, maintaining transfusion safety, and maintaining high blood quality [5]. Regular and voluntary blood donors can guarantee a reliable and adequate blood supply.

During the COVID-19 pandemic, the global city lockdown and social distancing measures seriously affected blood donation practice and further impacted blood transfusion services for patients in need in hospitals [6], and over the past few years, blood donation has become increasingly imperative. Only healthy individuals are eligible to donate blood during the pandemic [7]. Evidence shows that the main motivations for blood donation include altruism and a sense of responsibility [8]. Donors are motivated by a desire to save lives and help others, including family and friends [9], and many feel a duty to support their community and homeland [10], highlighting the intrinsic motivations donors perceive. Unfortunately, the pandemic significantly changed donation practices [6,11], notably reducing the intentions of donors and non-donors to donate blood [6,12], leading to an insufficient blood supply [13]. At the onset of the pandemic, active donors initially demonstrated greater awareness of their role in donating blood than before, whereas inactive donors reported reduced donation intention [12]. However, as the pandemic continued, both active and inactive blood donors became less willing and less morally compelled to contribute.

A systematic review of 98 studies focused on the impact of pandemics, mainly COVID-19, and natural disasters, primarily earthquakes, on blood supply. It found that blood donations decreased during the COVID-19 pandemic but increased after earthquakes [14], and the number of first-time donors was lower post pandemic compared to after earthquakes [14]. Another systematic review of 15 studies from different countries reported an overall 25% reduction in blood donation compared to the pre-COVID-19 pandemic period [6]. However, the rate varied across areas, ranging from a 71% decrease to a 10% increase after rigorous blood donation strategies [6], for instance, a 67% reduction in whole blood donors in China [15] and a decrease in the number of first-time donors [6]. Given that people were afraid of contracting the coronavirus, virtual schooling reduced school blood drives and other blood donation activities, further decreasing blood donation intentions [2]. The pandemic did not substantially increase blood demand, but it led to significant supply shortages due to a decline in available blood donors [15]. Evidence showed that most donors were older, returning donors, rather than new, young donors, during the pandemic [15]. Thus, promoting and maintaining an adequate blood supply, especially from young and first-time donors, is a crucial healthcare policy for every country worldwide.

In recent years, research has explored blood donation intentions and barriers among young adults and university students in different countries [9,10,16,17,18,19]. The blood donor rate has ranged from 15% to 33.6% in the past few years [9,16,17,18,19]. The primary obstacles identified include medical ineligibility or disqualification for blood donation [10,17,19], concerns about the risk of infectious disease transmission [9,10,18,19], never having been asked to donate blood [9,17], a lack of knowledge or misconceptions about the donation process [9,18,20], and donation centers that are difficult to access [19]. Apart from motivation and barriers to donating blood, studies also reported that knowledge is a crucial factor influencing blood donation intention [9,17,21]. Most university students have limited knowledge of blood donation [9,10], but it increases with blood donation experience [5,20,22] and with age [19,22].

Nursing students are future nursing professionals and should have sufficient knowledge and a professional obligation to provide health education and promote blood donation to the general public. They are also potential young blood donors who have a social responsibility to support a sufficient blood supply for lifesaving. Studies have shown that the blood donor rate among nursing students has ranged from 21.8% to 37.1% in the past few years across different countries; for example, it was 21.8% [23] and 26.2% [22] in Portugal, 34.8% in Italy [24], and 37.1% in Spain [22]. Casal-Otero et al. [23] reported low knowledge of blood donation eligibility requirements and highlighted that an insufficient understanding of the eligibility criteria may lead individuals to mistakenly believe that they cannot donate blood. Martínez-Santos et al. [22] reported that the knowledge of nursing students was lower than expected, and efforts should focus on targeted training and educational activities to raise knowledge and awareness and encourage blood donation. Cicolini et al. [24] found that blood donors had higher knowledge than non-donors. Most participants (81.5%) viewed their role as future healthcare professionals to educate and influence potential donors and to effectively motivate other students across various faculties and the university [24].

Hong Kong is not unique in this global imbalance in blood availability. Blood donation in Hong Kong is voluntary, non-remunerated, and anonymous, and the Hong Kong Red Cross Blood Transfusion Service collects blood and supplies it to all public and private hospitals in Hong Kong for clinical transfusion [25]. Nevertheless, blood collections have decreased by 14% over the past 10 years, with 219,200 units collected in 2024, which is below the Hong Kong Red Cross Blood Transfusion Service’s annual goal of approximately 237,900 units [26]. This decline was due to various factors, including the COVID-19 pandemic, post-pandemic increases in travel, and the postponement of donations after returning from areas with endemic mosquito-borne diseases. According to the Hong Kong Red Cross Blood Transfusion Service [27], blood collection dropped substantially from 4074 in the first 5 weeks of 2019 to 2362 in the first 5 weeks of 2020. In addition, the Government of the Hong Kong Special Administrative Region (HKSAR) urged the public to donate blood due to an insufficient daily supply to meet demand from local hospitals during the fifth wave of the local COVID-19 epidemic in 2022 [28]. As reported by the Legislative Council Secretariat of HKSAR in 2025, the blood donation rate in Hong Kong was 1.7% of the total population, lower than those in Japan (4%), South Korea (2.4%), and Australia (2.2%) as compared with other developed countries [26]. Additionally, the gradual decline in the new donor rate from 15.3% in 2014 to 9.4% in 2024 led to a 5.9% drop. Apart from a decrease in new donors, the ratio of young donors (aged 16–30) dropped from 45% to 17%, underscoring the need to recruit more young donors in the near-future.

In the last decade, immediately before the COVID-19 pandemic, only a few local studies examined the behavior and knowledge of young people regarding blood donation in Hong Kong [5,29]. Hong and Loke [29] investigated 3316 young adults aged 18 to 25 to identify factors influencing blood donation behavior. Most of these young individuals (76%) are blood donors, and about half of them reported donating blood for blood tests (53.1%) or for complimentary physical examinations (47.3%). Most blood donors viewed blood donation as a social responsibility (83.3%) and a moral duty (64.9%). Suen et al. [5] focused on 542 undergraduate university students aged 18 to 30 to assess their motivation and knowledge regarding blood donation, and 49.4% of them were blood donors. Most participants (61.7%) were unaware that breastfeeding women cannot donate blood, while half (50.8%) mistakenly believed that a woman’s menstrual period disqualifies her from donating. Furthermore, previous experience with blood transfusion and organ donor registration was a strong predictor of blood donation behavior. Both studies found that blood donors possessed greater knowledge of blood donation than non-blood donors [5,29].

Given the limited prior research on local nursing students and their dual roles and responsibilities, we aimed to investigate their knowledge and motivations regarding blood donation in Hong Kong following the COVID-19 pandemic. By exploring their understanding and motivations toward blood donation, we can improve their engagement, thereby increasing donor recruitment and ultimately mitigating blood shortages. Additionally, the insights gained can help develop strategies to empower nurses to contribute effectively to blood donation efforts.

## 2. Materials and Methods

### 2.1. Study Design

We used a cross-sectional, descriptive, quantitative design because of its efficiency in recruiting participants aligned with this study’s objectives. It allowed us to describe populations, examine the associations between variables, and identify trends and relationships, but it is challenging to establish causal relationships from cross-sectional analysis [30].

### 2.2. Participants

The target participants in this study were local university students aged 18 or older enrolled in full-time undergraduate general nursing programs who could read Chinese, as the questionnaire was in Chinese. The nursing students in mental health programs were excluded as they have a distinct curriculum and community role focused on mental health issues, which differs from that of general nursing students, such as blood donation promotion.

### 2.3. Sampling

Participants who were nursing students were recruited through convenience sampling from a large, self-funded, non-profit university, which offers both undergraduate and postgraduate programs and has one of the highest enrollments of general undergraduate nursing students in Hong Kong. The sample size was estimated using Slovin’s formula [31] with a known population size and an estimated margin of error. Given an estimated population size of nursing students of around 10,000 in Hong Kong and a margin of error of 0.05, a sample size of at least 370 would be required. The formula that was used to calculate the sample size was n = N/(1 + Ne^2^), where n is the sample size, N is the population size (N = 10,000), and e is the margin of error (if 5%, e = 0.05).

### 2.4. Instrument and Outcome Measures

In this study, we utilized a comprehensive online questionnaire divided into four sections to assess the knowledge and attitudes of general nursing students toward blood donation. The use of the Knowledge Questionnaire on Blood Donation [5] and the Blood Donor Identity Survey [5,32] in the questionnaire was approved by the authors.

The first section of the questionnaire collected demographic data, including gender, age, program year, religion, self-evaluated health status (e.g., any chronic illness, family medical history, blood transfusion), clinical experience, and part-time job experience, using binary yes/no questions.

The second section concerned the blood donation experience and included seven questions. Six nominal questions were collected to differentiate between non-blood donors and single/repeated/regular/rejected blood donors (with the reasons for denied blood donations). The last question was about the latest blood donation date for donors.

The third section of the questionnaire assessed the knowledge and understanding of participants about blood donation. We utilized the well-validated Knowledge Questionnaire on Blood Donation [5], which demonstrated excellent content validity and reliability, confirmed by a content validity index of 95% and an intraclass correlation coefficient of 0.914 [5]. It consisted of 12 nominal questions, with each correct answer earning 1 point and each incorrect answer receiving 0, resulting in a maximum possible knowledge score of 12. Higher scores indicate greater knowledge about blood donation. For example, questions included “Can pregnant women donate blood?” and “Are there age restrictions for blood donors?”

Lastly, the fourth section, adapted from the Blood Donor Identity Survey [32], assessed the motivations of participants to donate blood and the factors influencing their decisions, grounded in Self-Determination Theory (SDT). SDT provides a framework for understanding human motivation based on three innate psychological needs: autonomy, competence, and relatedness [33]; these needs are fundamental to motivation, personal growth, and overall well-being. The original survey, established by [32], was translated into a Chinese version by [5]. The survey consisted of six motivational factors, ranging from unmotivated to autonomous motivation to donate: Amotivation, External Regulation, Introjected Regulation, Identified Regulation, Integrated Regulation, and Intrinsic Regulation. The 18 survey items used a 7-point Likert scale ranging from 1 (strongly disagree) to 7 (strongly agree), and the scoring range of each subscale was 3–21. To assess the reliability of the translated Chinese survey [5], the internal consistency of the motivation subscales was examined, yielding Cronbach’s α values ranging from 0.7 to 0.9, which indicates an acceptable to excellent level of reliability. Additionally, content validity was confirmed by a panel of five experts, with an index of 93.3%.

### 2.5. Ethical Considerations

Ethical approval was obtained from the Research and Ethics Committee of Saint Francis University before the study’s implementation (Reference No. SEWG/HP/2023/Group 38) on 24 October 2023, and the research was conducted in accordance with the Declaration of Helsinki. Participation in the survey was entirely anonymous, and potential participants were fully informed about the study’s purpose and procedures. An information sheet was provided, and an electronic consent form was obtained before completing the online questionnaire. There were no significant risks associated with the study. Participants were assured that they could withdraw from the study at any time without it affecting their academic performance. All data were collected confidentially via online surveys and were accessible only to the research team members. The data will be deleted 5 years after completion of the study.

### 2.6. Data Collection and Analysis

Data collection took place in November 2023, following the approval of the research and ethics committee. General nursing students at the selected university were recruited after their on-campus classes. Before beginning the online survey, the research team provided a comprehensive explanation of the study to all potential participants. This explanation included the study’s aims, risks, benefits, procedures, and the voluntary nature of participation. Participants were encouraged to ask questions and express any concerns.

Online questionnaires, along with information sheets and consent forms, were distributed using a QR code that was linked to Google Forms. Those who did not consent to participate could not proceed to start the online questionnaire. By late 2023, approximately 2200 general nursing students were enrolled at the university, 711 of whom participated in the online survey. Of these, 650 students completed the survey, while 61 had incomplete or invalid entries and were excluded from the analysis, yielding a low response rate of about 29.5%.

The demographic characteristics of the participants are summarized using descriptive statistics. Normality of continuous data was assessed by the Shapiro–Wilk test. To assess significant differences between blood donors and non-blood donors, an independent-samples *t*-test was used when the continuous variables were normally distributed. For categorical data, the Pearson Chi-square test or Fisher’s Exact test was used to determine statistically significant differences across groups.

Variables demonstrating a marginal association (*p* < 0.1) in the univariate analyses were subsequently included in a logistic regression model to identify significant factor(s) related to blood donor status. Multicollinearity was checked by the Variance Inflation Factor (VIF), and VIF < 5 indicated no multicollinearity problem [34]. All statistical analyses were conducted using IBM SPSS Statistics for Windows, Version 26.0 (IBM Corp., Armonk, NY, USA) [35]. The threshold for statistical significance was set at two-tailed *p* < 0.05.

## 3. Results

Table 1 summarizes the characteristics of the participants and their association with blood donor status. The total sample size for the analysis was 650, consisting of 465 (71.5%) non-blood donors and 185 (28.5%) blood donors. Overall, 484 participants (74.5%) were female, and 166 (25.5%) were male, with a mean age of 20.84 ± 2.26. The univariate analysis showed that several factors differed significantly between the two groups. Blood donors were significantly older (21.63 ± 2.60) than non-donors (20.53 ± 2.03), with *p* < 0.001. Prior experience of receiving blood was one of the most distinguishing characteristics, with 35 blood donors (18.9%) having prior experience, compared to only 15 non-donors (3.2%) (*p* < 0.001). Similarly, 174 blood donors (94.1%) reported knowing someone who had donated blood, significantly higher than the 377 non-donors (81.1%), with *p* < 0.001. Conversely, taking medication was less common among blood donors (3.2%) than non-donors (9.2%; *p* = 0.009). Finally, having a religious affiliation (*p* = 0.001), any clinical experience (*p* = 0.001), ever having a part-time temporary nursing job (*p* = 0.012), being refused for blood donation (*p* = 0.005), and a higher score of self-report health status (*p* = 0.045) were associated with a higher chance of being a blood donor.

Table 2 compares the knowledge of participants regarding blood donation by donor status, with scores ranging between 3 and 12. Overall, blood donors (9.77 ± 1.28) demonstrated a significantly higher total knowledge score than non-blood donors (9.46 ± 1.33), with *p* = 0.006. The highest overall correct responses for all participants were “Hepatitis B carriers can donate blood” (98.0%) and “All patients with cancer with the risk of metastasis can donate blood” (95.5%), while the lowest correct responses were “Woman with menstruation can donate blood” (37.7%) and “Breastfeeding woman can donate blood” (51.5%).

A significantly higher percentage of blood donors (47.0%) correctly knew that a woman with menstruation can donate blood compared to non-blood donors (34.0%) (*p* = 0.002). Blood donors displayed a significantly higher percentage of correct answers regarding the deferral criterion that a person having a fever on the day of donation cannot donate blood (97.3% for donors versus 92.7% for non-donors; *p* = 0.026). A significantly higher percentage of blood donors (75.1%) correctly knew that a smoker can donate blood compared to non-blood donors (62.4%) (*p* = 0.002).

Table 3 displays the comparison of mean scores on the SDT subscales of motivation between blood donors and non-blood donors.

Compared with the non-donor group (11.61 ± 3.28), blood donors reported a significantly lower mean score on Amotivation (9.60 ± 2.69), with *p* < 0.001. Blood donors scored significantly higher on another three subscales: Identified Regulation (15.34 ± 3.46 vs. 13.95 ± 3.55, *p* < 0.001), Integrated Regulation (13.64 ± 3.56 vs. 12.02 ± 3.73, *p* < 0.001), and Intrinsic Regulation (13.68 ± 3.40 vs. 12.08 ± 3.44, *p* < 0.001).

The logistic regression model (Table 4) provided a satisfactory fit to the data by the Hosmer–Lemeshow goodness-of-fit test (*p* = 0.953). Seven variables were identified as significant factors of blood donor status. The values of VIF were between 1.05 and 4.66.

Prior experience of receiving blood (OR = 5.81, 95% CI: 2.92–11.54, *p* < 0.001), older age (OR = 1.22, 95% CI: 1.12–1.33, *p* < 0.001), knowing someone who had donated blood (OR = 2.63, 95% CI: 1.29–5.35, *p* = 0.008), and having a religious affiliation (OR = 1.81, 95% CI: 1.07–3.06, *p* = 0.028) were found to be significant factors associated with higher chances to donate blood. Conversely, taking medication was found to be a significant factor associated with a lower likelihood of being a blood donor (OR = 0.24, 95% CI: 0.09–0.63, *p* = 0.004). Within the ratings of donor identity, higher Amotivation scores reduced the odds of blood donation (OR = 0.83, 95% CI: 0.78–0.89), *p* < 0.001), while an increased score on Identified Regulation significantly and positively related to donor status (OR = 1.15, 95% CI: 1.09–1.22, *p* < 0.001).

## 4. Discussion

In our study, we aimed to investigate nursing students’ knowledge and motivations regarding blood donation in Hong Kong post pandemic. We surveyed 650 general nursing students from a single institution and found that only 185 (28.5%) were blood donors at the time of data collection in 2023, following the pandemic. Participants showed moderate knowledge (9.55 ± 1.32 out of 12) about blood donation. Nursing student participants who are older, have a religion affiliation, have previously received blood, know someone who has donated blood, and possess a higher score in Identified Regulation are more likely to become blood donors (odds ratio > 1). Conversely, nursing students who are on medication and have a higher score in Amotivation are less likely to donate blood (odds ratio < 1).

In recent years, studies have reported blood donation rates among nursing students in different countries, e.g., 21.8% [23] and 26.2% [22] in Portugal, 34.8% in Italy [24], and 37.1% in Spain [22]. In our study, we reported a 28.5% blood donation rate among nursing student participants following the COVID-19 pandemic in 2023. Wang et al. [15] recommended implementing effective educational programs to enhance young adults’ sense of social responsibility and accountability regarding blood donation.

In our study, older age was a significant predictor of greater blood donation motivation, consistent with previous studies [5,17,19,36]. In addition, evidence from both overseas studies [10,17,21,36] and local statistics [26] indicates a gender disparity in blood donation behavior, with males donating blood more frequently than females [10,17,26,36]. The key motivators for men to donate blood products include a desire to help others, a favorable view of incentives, health check-ups, and social expectations [37]. A local study reported contradictory findings that female young adults are more motivated than males to donate blood [5]. However, in our study, we did not identify any gender differences in blood donation practices.

Additionally, our study identified prior experience with blood transfusion and knowing someone who has donated blood as significant factors in blood donor status. Consistent with Suen et al.’s study [5], individuals who have previously received blood are generally more inclined to donate blood in appreciation of the kindness they have experienced from others. In our study, participants were refused blood donation (11.2%, n = 73) mainly due to physical or medical conditions. However, past experiences of rejection may not influence a person’s commitment or dedication to becoming a donor when they qualify [5]. Conversely, nursing students who are on medication were found to have a lower likelihood of being a blood donor in our study. Although it is not exactly that all individuals on regular medication have low motivation for blood donation, instead, the eligibility is based on the types of medication and medical conditions [38]. It most likely leads to a one-week deferral, but some may still donate after evaluation.

Among religiously affiliated participants, we found that those with religious affiliations were significantly more motivated to donate blood. This aligns with findings from Martinez et al. [39], which supported the connection between religiosity and the intention to donate blood. Their research on postgraduate health students indicated that those with religious beliefs who regularly donated blood showed higher levels of intrinsic religiousness than those who donated infrequently. By contrast, Zucoloto et al. [16] showed that Brazilian health sciences undergraduate students without religious beliefs donated blood less frequently. They emphasized that religious beliefs play a crucial role in influencing health decisions, promoting treatment adherence, and raising awareness of the importance of organ and blood donations in saving lives. To increase blood donation motivation, the local government and the Hong Kong Red Cross Blood Transfusion Service may consider collaborating with religious organizations to conduct blood donation promotion activities for their followers, thereby encouraging more followers to donate blood to benefit those in need in the near future.

Our participants demonstrated moderate knowledge about blood donation, achieving an average total score of 9.55 ± 1.32 out of a possible 12 (see Table 2), which is similar to the results of a local study [5]. The moderate level of knowledge is most likely achieved through the healthcare professional background gained through academic study and clinical practice, unlike other studies reporting low levels of knowledge among university students in different countries [9,10,19,23]. A multicenter survey of 12,606 university students from 16 countries found that knowledge about blood donation was limited, with only 28.5% reporting a good understanding. Students in health science programs showed significantly greater awareness than those in other fields [19]. Almost all of our participants (98%) correctly recognized that individuals with Hepatitis B cannot donate blood, as Hepatitis B is a prevalent infectious disease in Hong Kong and can be spread through contaminated blood or bodily fluids entering another person’s body [40]. While most of our participants had a certain understanding of blood donation, a significant number still held misconceptions about certain aspects. Half of the participants (51.5%) misbelieved that breastfeeding women could donate blood. Blood loss during labor can reduce iron levels [38]. Therefore, postpartum women with a history of blood loss should wait at least six months before donating blood to maintain adequate iron stores. More comprehensive, in-depth learning about blood donation is required to address the notable gaps.

Additionally, blood donors had a significantly higher total knowledge score than non-blood donors, consistent with other studies [5,22,24,36]. Although the magnitude of this difference was small in our study, it is educationally meaningful. For instance, a significantly higher percentage of blood donors (47.0%) correctly knew that a woman who is menstruating can donate blood compared to non-blood donors (34.0%) (*p* = 0.002). Indeed, menstruation does not affect eligibility to donate blood [41], but menstruating women should ensure that they get adequate sleep before donating and rest afterward. If they experience pain or feel unwell, they should consider postponing their donation. Similarly, more blood donors than non-donors (97.3% vs. 92.7%) recognized that individuals cannot donate blood if they have a fever. Donors may donate blood only after recovery from the disease and resolution of fever [38]. Misunderstanding the blood donation eligibility criteria may lead individuals to mistakenly believe that they are ineligible to donate blood, consistent with the study by Casal-Otero et al. [23]. It is recommended that nursing education and knowledge about blood donation be enhanced, particularly regarding women’s physiological status and donors’ health, and that outreach to the general public be expanded.

According to Self-Determination Theory, the behavior of individuals depends more on internal than on external motivation [32]. The self-determination continuum ranged from a complete lack of motivation (Amotivation) to enjoyment and personal identification (Intrinsic Regulation). For blood donors, whether first-time or repeat, individual values, prosocial motivation, and ease of access are primary motivators [42]. By contrast, unpleasant blood donation experiences, low self-efficacy for donating, concerns about access, unappealing incentives, and inadequate knowledge about blood donation were common deterrents for both blood donors and non-blood donors [42]. In our study, 90 of 185 blood donors (48.6%) donated only once, whereas only 29 (4.5%) were regular contributors. With respect to donor identity results in our study, the significant factors of “Amotivation” and “Identified Regulation” indicated that non-donors and donors viewed blood donation as having no intention to donate, and as a personally significant and valuable choice to donate for others’ health, respectively [32]. To establish a culture of giving and helping others, institutional and educational curricula in nursing programs are suggested to incorporate training on the importance of blood donation.

Ensuring a safe and adequate blood supply through regular voluntary, non-remunerated blood donations is crucial for health systems worldwide [1]. A systematic review of 23 studies on improving first-time donor retention found that positive attitudes and a sense of control promote retention, whereas anxiety, negative experiences, and deferrals act as barriers [43]. Though common strategies such as reminders and incentives are implemented, their effectiveness in encouraging first-time donors to return is often limited. Similar concepts are discussed in the study by Li et al. [44], which found that neither intrinsic nor extrinsic rewards significantly influence the intention of donors to donate blood again in China. Additionally, fear negatively affects this intention. Major reasons for not donating blood are concerns about the safety of blood collection [20] and the risk of contracting an infectious disease during donation [36]. Therefore, strategies should focus on addressing donors’ fears rather than just appealing to rewards.

The COVID-19 pandemic substantially affected blood donation intentions among both blood donors and non-blood donors, particularly reducing medium- and long-term donation intentions [12]. Unlike other disasters or crises, the COVID-19 pandemic threatened the health of citizens across society and affected blood donation behavior in distinct ways. Veseli et al. [12] reported that although active donors increased their blood donation in the early stages of the pandemic, this trend reversed as the pandemic progressed. A possible reason was the erosion of moral norms and personal self-efficacy to comply with societal legal policies, such as social distancing measures and work-from-home [12]. The authors emphasized the significance of blood donor recruitment and retention during the pandemic.

Another study conducted at the beginning of the pandemic in China reported a 72.7% reduction in young blood donors (aged 18–25) and a 49.3% increase in donors aged 45 or older [15]. This shift may be attributed to parental disapproval of going out during lockdown and a lack of motivation and commitment among younger people. However, the number of older regular donors increased by 57%, ensuring a sufficient blood supply to meet urgent needs [15], which echoed Veseli et al.’s study mentioned above. Siu et al. [45] investigated the motivations and deterrents to blood donation among individuals aged 18 to 65 in Hong Kong. They found that perceptual, social, and institutional factors significantly influenced the motivation of blood donors to donate during the pandemic [45]. Pre-existing social factors also significantly influenced non-donors and lapsed donors. Quarantine measures and new social norms discouraged participation, while tensions between mainland China and Hong Kong and negative experiences at donor centers persisted. To encourage donations, it is essential to tackle both pandemic-related and pre-existing barriers [46]. There is currently a concerning decline in the recruitment of new and young blood donors in Hong Kong. From 2014 to 2024, the rate of new donors dropped by 6%, while the number of young donors aged 16 to 30 decreased by 28% [26]. These trends underscore the significant challenges in attracting new young donors.

### 4.1. Implications for Practice and Policy

To address the aforementioned knowledge and motivation gaps regarding blood donation, collaboration among the Hong Kong government, healthcare organizations, and university nursing faculties is essential. Together, they can develop strategies to promote blood donation among future nursing professionals and the general public. Increasing awareness through expanded blood donation campaigns and online platforms can help educate young people, dispel misconceptions, reduce fear and anxiety about blood donation, and nurture a sense of social responsibility.

Additionally, extending donor center opening hours and deploying mobile donor units would increase access to blood donation. Partnering with local universities to organize regular blood drives and campus awareness campaigns can further disseminate accurate information about blood donation.

Nursing curricula should incorporate training on the importance of blood donation to help foster a culture of giving. Nursing students, as future healthcare professionals, also have a professional obligation to provide health education and promote blood donation to the public. By collaborating with religious groups or local community organizations, nursing students can also serve as public educators to promote blood donation and donor recruitment. This will help ensure a steady supply of safe blood for those in need in the near future. Future in-depth research on strategies or interventions to facilitate blood donation or a longitudinal study to evaluate the transition from blood donation intention to behavior is also suggested.

### 4.2. Limitations

When interpreting this study’s results, it is important to acknowledge certain limitations and potential biases. First, this study’s cross-sectional descriptive design limits causal interpretations and long-term insights into blood donation trends and the study population’s characteristics. A mixed-methods longitudinal study examining participants’ barriers and motivations to blood donation would also be valuable in the near future.

Second, the convenience sampling in this study limited the representativeness and raised concerns about bias, thereby affecting the interpretation and generalizability of the findings. While this single, non-profit university, with about 2200 nursing students in 2023, offers diverse programs, its student demographics may not reflect the broader nursing student population of around 10,000 in Hong Kong. In addition, the low response rate may cause nonresponse bias. Nonetheless, the insights gained can help improve blood donation rates and increase knowledge among healthcare professionals and the public in the future. A future multi-site study can use random sampling to include students from various educational settings, thereby reducing sampling bias.

Third, data were collected via an online self-report questionnaire administered to nursing students. While this method is efficient and cost-effective, it raises concerns about the validity and reliability of the responses [47]. Participants voluntarily accessed the questionnaire via a QR code after their classes. Although online questionnaires can introduce bias or lead to misunderstandings, participants had the opportunity to ask clarifying questions.

Finally, self-reporting and recall bias regarding blood donation and health history can create significant limitations for this study. The memories of participants may lead to inaccuracies instead of valid clinical data. Moreover, self-report surveys may initiate social desirability or response bias, affecting the validity and accuracy of the measurements [48]. A future study that includes clinical data, such as donation records, is suggested.

## 5. Conclusions

Blood donation rates among local nursing students were low following the pandemic, despite moderate overall knowledge. Blood donors exhibited significantly higher knowledge than non-donors. Although moderate knowledge was observed, strengthening nursing education and knowledge about blood donation, particularly regarding women’s physiological status, is recommended. Factors that increased the likelihood of donating included age, religion, prior blood receipt, knowing a donor, and higher Identified Regulation scores. Conversely, students on medication and those with higher Amotivation scores were less likely to donate. Collaboration among the Hong Kong government, healthcare organizations, and university nursing faculties is vital to promote blood donation among future nursing professionals and the public. Increasing awareness through campaigns and online platforms can educate young people, dispel misconceptions, and foster social responsibility. Additionally, extending donor center hours and introducing mobile units would improve accessibility. Partnering with local universities to host regular blood drives and run awareness campaigns can further spread accurate information about blood donation. Through effective educational programs, the sense of accountability of young adults regarding blood donation can be enhanced, while engaging with religious organizations can also promote awareness and encourage donations.

## Figures and Tables

**Table 1:** Characteristics of participants related to donating blood.

Variable	Total (N = 650)	Non-Blood Donor (N = 465)	Blood Donor (N = 185)	OR (95% CI)	*p*-Value
Gender—female	484 (74.5%)	340 (73.1%)	144 (77.8%)	1.29 (0.86–1.93)	0.213
Age (years)	20.84 ± 2.26	20.53 ± 2.03	21.63 ± 2.60	1.23 (1.14–1.33)	* <0.001
Study year					0.094
Year 1	196 (30.2%)	152 (32.7%)	44 (23.8%)	1	
Year 2	151 (23.2%)	108 (23.2%)	43 (23.2%)	1.38 (0.85–2.24)	
Year 3	132 (20.3%)	93 (20.0%)	39 (21.1%)	1.45 (0.88–2.39)	
Year 4	83 (12.8%)	51 (11.0%)	32 (17.3%)	2.17 (1.24–3.78)	
Year 5	88 (13.5%)	61 (13.1%)	27 (14.6%)	1.53 (0.87–2.69)	
Religion	89 (13.7%)	50 (10.8%)	39 (21.1%)	2.22 (1.40–3.51)	* 0.001
Chronic illness	43 (6.6%)	32 (6.9%)	11 (5.9%)	0.86 (0.42–1.74)	0.665
Chronic illness (parents)	247 (38.0%)	173 (37.2%)	74 (40.0%)	1.13 (0.79–1.60)	0.508
Have medication	49 (7.5%)	43 (9.2%)	6 (3.2%)	0.33 (0.14–0.79)	* 0.009
Regular follow-up	83 (12.8%)	66 (14.2%)	17 (9.2%)	0.61 (0.35–1.07)	0.085
Know someone who has ever received the blood	202 (31.1%)	147 (31.6%)	55 (29.7%)	0.92 (0.63–1.33)	0.640
Prior experience of receiving blood	50 (7.7%)	15 (3.2%)	35 (18.9%)	7.00 (3.72–13.18)	* <0.001
Any clinical experience	184 (28.3%)	115 (24.7%)	69 (37.3%)	1.81 (1.26–2.61)	* 0.001
Any part-time work	476 (73.2%)	331 (71.2%)	145 (78.4%)	1.47 (0.98–2.20)	0.062
Ever part-time temporary nursing job	147 (22.6%)	93 (20.0%)	54 (29.2%)	1.65 (1.12–2.44)	* 0.012
I was refused for blood donation	73 (11.2%)	42 (9.0%)	31 (16.8%)	2.03 (1.23–3.34)	* 0.005
Know someone who had donated blood	551 (84.8%)	377 (81.1%)	174 (94.1%)	3.69 (1.92–7.09)	* <0.001
Self-report health status (0–100)	65.28 ± 15.31	64.52 ± 15.57	67.19 ± 14.51	1.01 (1.00–1.02)	* 0.045

* *p* < 0.05. Continuous variables are presented as mean ± SD and analyzed by the independent-samples *t*-test. Categorical variables are presented as N (%) and analyzed by the Pearson Chi-square test or Fisher’s Exact test.

**Table 2:** Comparison of knowledge regarding blood donation by donor status.

Items	Total (N = 650)	Non-Blood Donor (N = 465)	Blood Donor (N = 185)	
	N (%)/Mean ± SD	N (%)/Mean ± SD	N (%)/Mean ± SD	*p*-Value
Total knowledge score	9.55 ± 1.32	9.46 ± 1.33	9.77 ± 1.28	* 0.006
1. All donated blood being tested for the presence of HIV antibodies				
Yes #	546 (84.0%)	395 (84.9%)	151 (81.6%)	0.297
No	104 (16.0%)	70 (15.1%)	34 (18.4%)	
2. Person under 16 years can donate blood				
Yes	169 (26.0%)	122 (26.2%)	47 (25.4%)	0.827
No #	481 (74.0%)	343 (73.8%)	138 (74.6%)	
3. Pregnant woman can donate blood				
Yes	121 (18.6%)	91 (19.6%)	30 (16.2%)	0.322
No #	529 (81.4%)	374 (80.4%)	155 (83.8%)	
4. All people with diabetes or hypertension can donate blood				
Yes	61 (9.4%)	44 (9.5%)	17 (9.2%)	0.914
No #	589 (90.6%)	421 (90.5%)	168 (90.8%)	
5. All patients with cancer with the risk of metastasis can donate blood				
Yes	29 (4.5%)	19 (4.1%)	10 (5.4%)	0.462
No #	621 (95.5%)	446 (95.9%)	175 (94.6%)	
6. Woman with menstruation can donate blood				
Yes #	245 (37.7%)	158 (34.0%)	87 (47.0%)	* 0.002
No	405 (62.3%)	307 (66.0%)	98 (53.0%)	
7. There is an age limit for blood donation				
Yes #	601 (92.5%)	430 (92.5%)	171 (92.4%)	0.986
No	49 (7.5%)	35 (7.5%)	14 (7.6%)	
8. Breastfeeding woman can donate blood				
Yes	315 (48.5%)	227 (48.8%)	88 (47.6%)	0.774
No #	335 (51.5%)	238 (51.2%)	97 (52.4%)	
9. It is necessary for the donated blood to be used within 24 h				
Yes	67 (10.3%)	46 (9.9%)	21 (11.4%)	0.581
No #	583 (89.7%)	419 (90.1%)	164 (88.6%)	
10. Hepatitis B carriers can donate blood				
Yes	13 (2.0%)	11 (2.4%)	2 (1.1%)	0.368
No #	637 (98.0%)	454 (97.6%)	183 (98.9%)	
11. Person having fever on the day of donation can still donate blood				
Yes	39 (6.0%)	34 (7.3%)	5 (2.7%)	* 0.026
No #	611 (94.0%)	431 (92.7%)	180 (97.3%)	
12. Smoker can donate blood				
Yes #	429 (66.0%)	290 (62.4%)	139 (75.1%)	* 0.002
No	221 (34.0%)	175 (37.6%)	46 (24.9%)	

* *p* < 0.05. # correct answer. Continuous variables were analyzed by the independent-samples *t*-test, whereas categorical variables were analyzed by the Pearson Chi-square test or Fisher’s Exact test.

**Table 3:** Comparison of donor identity by motivations to donate blood.

Items	Total (N = 650)	Non-Blood Donor (N = 465)	Blood Donor (N = 185)		
	Mean ± SD	Mean ± SD	Mean ± SD	OR (95% CI)	*p*-Value
Amotivation	11.04 ± 3.25	11.61 ± 3.28	9.60 ± 2.69	0.81 (0.76–0.86)	* <0.001
External Regulation	8.74 ± 3.51	8.70 ± 3.46	8.83 ± 3.65	1.01 (0.96–1.06)	0.675
Introjected Regulation	8.89 ± 3.31	8.78 ± 3.26	9.15 ± 3.44	1.03 (0.98–1.09)	0.201
Identified Regulation	14.34 ± 3.58	13.95 ± 3.55	15.34 ± 3.46	1.13 (1.07–1.19)	* <0.001
Integrated Regulation	12.48 ± 3.75	12.02 ± 3.73	13.64 ± 3.56	1.13 (1.08–1.19)	* <0.001
Intrinsic Regulation	12.53 ± 3.50	12.08 ± 3.44	13.68 ± 3.40	1.15 (1.09–1.22)	* <0.001

* *p* < 0.05. Continuous variables were analyzed by the independent-samples *t*-test.

**Table 4:** Logistic regression identifying the factors of motivations to donate blood.

Variables	OR (95% CI of OR)	*p*-Value
Age	1.222 (1.122–1.332)	<0.001
Religion (reference group: no religion)	1.807 (1.067–3.059)	0.028
Have medication (reference group: no medication)	0.238 (0.089–0.632)	0.004
Prior experience of receiving blood (reference group: no prior experience of receiving blood)	5.808 (2.923–11.541)	<0.001
Know someone who had donated blood (reference group: did not know someone who had donated blood)	2.625 (1.288–5.350)	0.008
Amotivation	0.832 (0.776–0.891)	<0.001
Identified Regulation	1.151 (1.085–1.222)	<0.001

OR—odds ratio. CI—confidence interval. By Hosmer–Lemeshow goodness-of-fit: Chi-square = 2.674, df = 8, *p* = 0.953.

## Data Availability

The data presented in this study are available upon reasonable request from the corresponding author. They are not publicly available, due to privacy or ethical restrictions.
